# Exploring the neural underpinnings of chord prediction uncertainty: an electroencephalography (EEG) study

**DOI:** 10.1038/s41598-024-55366-1

**Published:** 2024-02-26

**Authors:** Kentaro Ono, Ryohei Mizuochi, Kazuki Yamamoto, Takafumi Sasaoka, Shigeto Ymawaki

**Affiliations:** 1 Center for Brain, Mind and KANSEI Sciences Research, Hiroshima University, Hiroshima, Japan; 2 Graduate School of Humanities and Social Sciences, Hiroshima University, Higashihiroshima, Japan

**Keywords:** Psychology, Human behaviour, Auditory system, Neuroscience, Cognitive neuroscience, Perception

## Abstract

Predictive processing in the brain, involving interaction between interoceptive (bodily signal) and exteroceptive (sensory) processing, is essential for understanding music as it encompasses musical temporality dynamics and affective responses. This study explores the relationship between neural correlates and subjective certainty of chord prediction, focusing on the alignment between predicted and actual chord progressions in both musically appropriate chord sequences and random chord sequences. Participants were asked to predict the final chord in sequences while their brain activity was measured using electroencephalography (EEG). We found that the stimulus preceding negativity (SPN), an EEG component associated with predictive processing of sensory stimuli, was larger for non-harmonic chord sequences than for harmonic chord progressions. Additionally, the heartbeat evoked potential (HEP), an EEG component related to interoceptive processing, was larger for random chord sequences and correlated with prediction certainty ratings. HEP also correlated with the N5 component, found while listening to the final chord. Our findings suggest that HEP more directly reflects the subjective prediction certainty than SPN. These findings offer new insights into the neural mechanisms underlying music perception and prediction, emphasizing the importance of considering auditory prediction certainty when examining the neural basis of music cognition.

## Introduction

Our perception of the external world is crucial for interacting with our environment, and making predictions enhances this ability. Predictive coding theory suggests that our brains constantly form predictions and correct prediction errors to optimize our understanding of the world^1,2^. This concept has also been applied to music perception, where prediction errors about musical notes elicit surprise and emotional reactions^3,4^. Several studies have reported the importance of predictions and prediction errors for remembering, creating, and emotionally responding to music^5–11^.

In Western music, predictable structures like rhythm and harmony shape our musical experience. Harmonically related chord sequences, known as chord progressions, are particularly important for forming the harmonic structure. Listeners prefer and expect such sequences, for example, the I-IV-V-I progression, which includes the tonic chord (I), the subdominant chord (IV), the dominant chord (V), and then returns to the tonic chord (I). While listening to such a chord progression, listeners gradually develop a musical context or harmonic structure like a tonal key, and the context helps predict subsequent chords^12^.

Electroencephalography (EEG) studies have reported that violation of chord prediction produces unique brain responses, such as early right anterior negativity (ERAN) and N5. These responses often manifest as negative potentials around 150–200 ms (for ERAN) and 500–700 ms (for N5) in the frontal area^13–24^, and their amplitudes increase over the course of a chord progression^25–27^. While ERAN is interpreted as indicator of prediction error in chord progression, N5 is suggested to reflect processes of harmonic integration, entailing a modification of listener’s hierarchy of harmonic stability^3,28^. However, the specific mechanism of generating harmonic predictions remains unclear, and the primary purpose of the present study is to elucidate the neural dynamics involved in generating harmonic predictions.

Stimulus Preceding Negativity (SPN) is an EEG component connected to predictive processes^29–32^. This component typically exhibits a negative shift in brain potentials while predicting a target stimulus. Studies have shown that SPN responds in an anticipation of a variety of stimuli, including symbolic pictures, beep sounds, and pain^33–37^, underscoring its role as a neural marker of predictive processing. SPN is usually defined by the mean amplitude during the last 200 ms before the target stimulus^38^. It is mainly observed in the right hemisphere, with its amplitude increasing as prediction uncertainty rises^31,34,35,39–41^. For instance, León-Cabrera and colleagues observed an increase in SPN amplitude corresponding to the unpredictability of the last words in sentences^42,43^.

While SPN has been a valuable tool in exploring sensory prediction at a neural level, its role in chord prediction remains unexplored. Given the effectiveness of SPN in evaluating predictive processes for various stimuli, including language, and the shared neural pathways between language and music^44–46^, we propose that SPN could also be a significant indicator for assessing chord prediction. Therefore, we hypothesize that the SPN amplitude is associated with uncertainty of chord prediction. Furthermore, since ERAN and N5 are recognized as indicators of the error of chord prediction and the subsequent harmonic integration process, it is plausible that SPN is similarly linked to these responses.

Recent research also emphasizes the importance of interoception, the processing and perception of internal bodily sensations (such as heartbeat, breathing, and others), in shaping various cognitive processes, based on the predictive coding perspective^47–51^. According to this view, interoceptive predictions help allocate internal resources in response to the external environment. For instance, functional states of the heart (systole or diastole) influence the perception and neural responses to sensory stimuli^52–59^.

Furthermore, the Heartbeat Evoked Potential (HEP), a neural response time-locked to heartbeats, is one potential method for examining the neural basis of interoceptive prediction. Though variations exist, a typical HEP characteristically peaks between 200 and 500 ms post-heartbeat and is primarily distributed in the frontal area^60^. Some studies have found that HEP prior to a visual or somatosensory stimulus can predict brain activity for these stimuli^59,61–63^, suggesting that the brain uses interoceptive signals to predict upcoming sensory (exteroceptive) signals. Based on these findings, we hypothesize that interoceptive signals, reflected by HEP, guide chord predictions, and that the HEP amplitude is associated with the certainty of chord prediction. A chord that was presented within a strong harmonic context is more predictable than those within a weak harmonic context. This predictability difference likely reaches its peak immediately before the final chord. This variance in predictability is expected to correspond with changes in HEP amplitude. Moreover, following previous studies, we expected a relationship between HEP amplitude and the ERPs elicited by the subsequent stimulus^59,61–63^. To investigate these issues, participants were presented with sequences of chords, both harmonically related and unrelated, and asked to predict the final chord. We expected that the degree of certainty in matching the predicted and actual chord progressions would associate with SPN and HEP amplitudes.

In summary, we propose that the certainty of chord prediction is linked to both SPN and HEP. Moreover, these responses may be connected to ERAN and N5, which reflect the prediction error. To examine these hypotheses, participants listened to harmonically appropriate chord progressions (I-IV-V-I) and randomly ordered, non-harmonic sequences of chords. They were instructed to predict the final chord. The brain potentials, measured using EEG, were compared between the harmonic and non-harmonic chord sequences during and after the prediction processes.

## Methods

### Participants

Twenty-seven participants (16 men and 11 women) with a range of individuals with 0 to 15 years of musical experience (mean ± standard deviation [SD]: 4.4 ± 5.3) between the ages of 19 and 32 (mean ± SD: 22.3 ± 3.0) were enrolled in this study. All participants were graduate and undergraduate students at Hiroshima University and right-handed, as determined by the Japanese version of the FLANDERS Handedness questionnaire^64^. None of the participants reported any motor, hearing, visual, or neurological deficiencies. Written informed consent was obtained from all participants before the experiment, which was conducted in accordance with the ethical standards of the Declaration of Helsinki. The study was also approved by the local ethics committee of Hiroshima University.

### Stimuli

**Figure 1:**
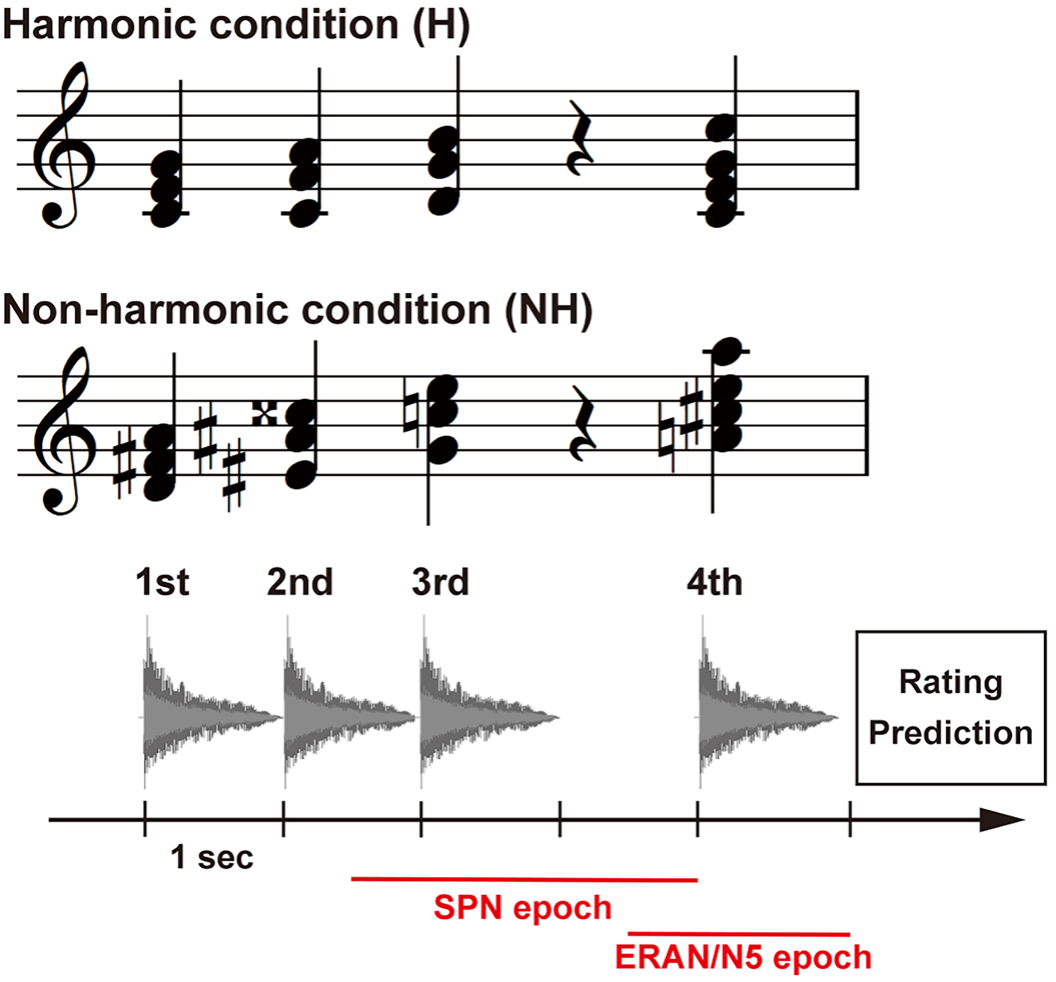
Example of stimulus presentation in a trial. **Top:** Example of a chord sequence. The harmonic condition consists of a harmonically appropriate chord progression in Western music theory, including the tonic (I), subdominant (IV), dominant (V), and tonic (I) chords. In the non-harmonic condition, each of the four chords is randomly selected from a pool of chords in twelve keys. For example, the first chord is chosen at random from the tonic chords in twelve keys. **Bottom:** Example of a trial. Each chord lasts 1 s, followed by a 1-s silent interval between the third and fourth chords. After the chord sequence is presented, a question regarding prediction certainty is displayed until the participant responds. Red colored horizontal lines indicate the epoch duration of SPN and ERAN/N5.

A sequence of four chords served as the stimuli, each generated by a piano sound (65 dB SPL) using Finale (version 26, MakeMusic, USA). Two distinct types of chord sequences were devised as stimuli. One sequence was designed as a popular chord progression, comprising the tonic, subdominant, dominant, and tonic (I-IV-V-I), in twelve major keys from C major to B major, referred to as a “harmonic condition”. The other sequence was defined as a “non-harmonic condition”. Each of the four chords in the non-harmonic condition was selected from a pool of chords in twelve keys for each chord function. That is, the first chord was randomly chosen from the tonics of the chord progression in twelve keys. The second chord was randomly chosen from the subdominants in twelve keys. The third and final chords were randomly chosen from the dominants and tonics in twelve keys, respectively. Examples of the harmonic and non-harmonic conditions are depicted in Fig. 1. Example of the non-harmonic condition consisted of the tonic in D major, the subdominant in E sharp major, the dominant in F major, and the tonic in A major. In each condition, the duration of each chord was one second, and a silent interval of one second was inserted between the third and final chords to prompt the participants to predict the final chord (Fig. 1 bottom).

### Procedure

Participants were seated in a chair in a dimly lit soundproof room. Participants sat in front of a 24-inch display (U2412M, 1920 × 1200 pixels, 60 Hz, Dell, USA) and listened to stimuli through in-ear earphones (Etymotic Research ER4, Etymotic Research Inc., USA). During each trial, as depicted in Fig. 1, a sequence of chords was presented, and during the silent interval between the third and final chords, the participants were asked to internally predict the final chord. After listening to the final chords, they were also asked to rate their “prediction certainty”–how well the progression from the third to the fourth chord matched their prediction. This was done using a Likert scale ranging from 1 (completely unmatched) to 10 (completely matched), with the ratings entered using the number keys (from 0 to 9) on the keyboard. Upon completing the rating, participants would immediately proceed to the subsequent trial. The experiment was divided into four sessions, each comprising 36 trials, presented in a random order of 18 chord sequences and 18 non-harmonic sequences. Participants were allowed to rest between sessions, and the experiment lasted approximately 45 min. All procedures were programmed using Presentation (ver. 20.0, Neurobehavioral Systems, Inc., USA) and performed using a Windows PC (DAIV Z9, Mouse Computer, Japan) with Realtek HD Audio.

### EEG measurement

EEG, electrocardiogram (ECG), and electrooculogram (EOG) signals were sampled at a rate of 1000 Hz using a BrainAmp DC amplifier (BrainProducts GmbH, Germany) and a 64-channel actiCAP electrode system. The electrodes were positioned according to the extended international 10–10 system, with the ground electrode located on the forehead (position: Fpz) and a nose reference. The electrode–skin conductance was kept below 10 kΩ. ECG was recorded with electrodes placed above the clavicle on the right side and in the region between the pelvis and rib on the left side. EOG were recorded with electrodes placed above and below the left eye (vertical EOG) and beside both eyes (horizontal EOG) to evaluate the eye movement artifact.

### EEG preprocessing

EEG data were analyzed using EEGLAB^65^. Initially, R peaks in the ECG were identified using a custom-made MATLAB script with the “findpeaks” function, and the timing of R peaks was employed as a trigger to extract HEP epochs from EEG data. Following down-sampling to 500 Hz and filtering with high-pass (0.1 Hz) and low-pass (40 Hz) filters, EEG data were re-referenced to the average montage. The time window between 200 ms preceding and 600 ms following the R peaks within a 2-s interval from the onset of the third chord to the end of the silent interval was extracted as an epoch for HEP analysis. The time window between − 500 and 2000 ms following the third chord was extracted as an epoch for SPN (Fig. 1 bottom). The decision regarding the width of this time window was informed by protocols established in previous SPN studies^30,33,36,66^. The reasons that we selected these time ranges as time window for HEP and SPN is described in the following section. For ERAN and N5, the time window between − 500 ms and 1000 ms after the fourth chord was extracted as epoch (Fig. 1 bottom). To eliminate noise artifacts from these epochs, independent component analysis (ICA) implemented in EEGLAB was conducted to separate independent components, and artifactual components were removed using the MARA plug-in^67,68^. Subsequently, epochs with peak-to-peak amplitudes exceeding 100 μV were eliminated as artifacts. The baseline was corrected with a time window between − 200 and 0 ms from the onset of the R peaks for HEP and between − 500 ms and 0 ms from the onset of the third chords for SPN and the fourth chords for ERAN and N5. Despite the baseline for SPN being distant from the SPN epoch (between 1800 and 2000 ms), we adhered to this approach as it was informed by previous SPN studies^30,33,36,66^.

### HEP analysis

After the preprocessing, 143 ± 25 (mean ± SD) trials were retained in the harmonic condition, and 145 ± 21 trials were retained in the non-harmonic condition. In general, HEP waveforms are commonly accompanied by cardiac-field artifacts synchronizing with the heartbeat^69^. Thus, HEP analysis needs to minimize the impact of such artifacts as much as possible. To this end, the HEPs were transformed into current source density (CSD) using the ERPLAB plug-in^70^. The EEG data recorded from scalp electrodes do not accurately reflect the specific activity of local brain sources but rather the “volume-conducted” activity. The CSD transformation acts as a high-pass spatial filter that minimizes volume-conducted contributions from distant regions and sources, including the cardiac-field artifacts^71,72^.

We selected frontal electrodes (left: F3, F5, AF3, and AF7; middle: F1, Fz, F2, and AFz; right: F4, F6, AF4, and AF8) as three regions of interest (ROI) following the frontal topography of the HEP in previous studies^55,73–76^. Though previous studies have shown typical HEP latency between 200 and 500 ms^55,77–80^, cardiac-field artifacts are most prominent during the QRS complex and the T wave of the heart cycle (~ 300 ms from the R peak)^81^. Consequently, we confined the time range to 300–500 ms following the R peaks to mitigate the impact of these artifacts as much as possible. The mean value of the HEP from 300 to 500 ms after the R peak was calculated, and a two-way analysis of variance (ANOVA) with factors of harmony (harmonic and non-harmonic) and ROIs (left, middle, and right) was conducted to compare the harmonic and non-harmonic conditions. Due to the presence of ceiling effects in participants’ subjective ratings, the correlation with their prediction certainty ratings was analyzed using Spearman’s rank correlation coefficient.

### ERP analysis

After the preprocessing, 66 ± 11 trials (92.6%) were retained in the harmonic condition, and 63 ± 12 trials (88.6%) were retained in the non-harmonic condition. Since applying the CSD transformation makes comparisons with previous studies more difficult, the SPN and ERAN/N5 were not transformed to CSD. Since SPN has often been reported with a right hemisphere dominance, and some studies have reported the SPN at the parietal area in this time window^32–34,66,82^, we selected target electrodes as P3 (right parietal) and P4(left parietal). The mean amplitude of SPN was analyzed using a two-way ANOVA with the factors of laterality (right and left) and harmony (harmonic and non-harmonic). Also, Spearman’s rank correlation coefficient was calculated to analyze the correlation between subjective ratings of prediction certainty and SPN.

The time window for ERAN was defined as between 150 and 250 ms, in line with prior studies^15,83,84^, and that for N5 was between 500 and 700 ms, as used in a previous study^85^. Additionally, the target electrodes for ERAN and N5 were determined as the right frontal electrodes (F6, F8, and AF8) in accordance with the previous ERAN/N5 studies. The mean amplitude of these ERPs was analyzed using a two-way ANOVA, with harmony (harmonic and non-harmonic) and laterality (left hemisphere [F5, F7, and AF7] and right hemisphere [F6, F8, and AF8]) as factors. Further, the correlation with subjective ratings of prediction certainty was analyzed using Spearman’s rank correlation coefficient.

### ECG control analysis

It is important to consider whether artifactual effects in heartbeats may have contributed to the observed results. Therefore, to assess whether the observed difference in HEP did not stem from a difference in ECG, we also analyzed ECG data between the onset of the third chord and the silent interval preceding the final chord. After down-sampling to 500 Hz and applying high-pass (0.1 Hz) and low-pass (40 Hz) filtering, we extracted the time window between 200 ms before and 600 ms after the R peaks as an epoch. Subsequently, we corrected for baseline with a time window between − 200 and 0 ms and compared the mean value of ECG between 300 and 500 ms between the harmonic and non-harmonic conditions using a paired *t*-test.

Since our experiment used popular chord progressions in Western music as stimuli, we expected that even participants without formal musical training would have been exposed to these stimuli since childhood. This exposure, we believe, minimized the potential impact of musical experience on the experiment’s results. Additionally, with geographical constraints, recruiting a sufficient number of participants with extensive musical experience proved challenging. Consequently, we did not divide participants into distinct groups of musicians and non-musicians. All statistical analyses were performed using R Studio (version 1.0.136) and R software (version 3.3.2). To address the multiple comparison problem, *p-*values were corrected using Bonferroni’s method. Partial *η*^2^ for ANOVA and *r* for *t*-tests were calculated as effect sizes.

## Results

### Behavioral data

**Figure 2:**
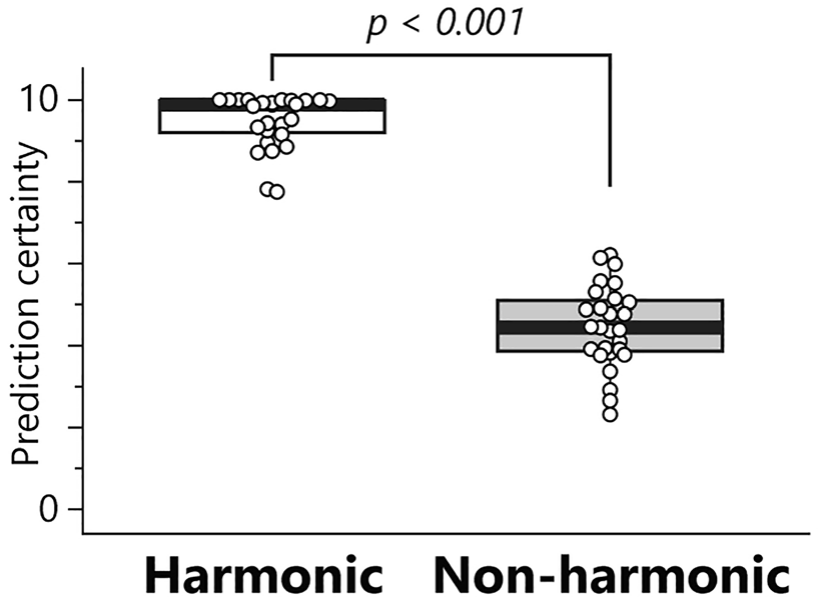
Subjective ratings of prediction certainty. The box-and-whisker plot displays the median, lower/higher quantile, and minimum/maximum of the individual data (depicted as white circles).

Figure 2 illustrates the subjective ratings regarding the prediction certainty across participants. The Wilcoxon signed-rank test revealed a significant difference between the sequences for the prediction certainty (*z* = 5.66, *p* < 0.001, *r* = 0.77). This result indicates that harmonically appropriate chord progressions predicted with higher certainty than randomly ordered non-harmonic chord sequences.

### EEG data

**Figure 3:**
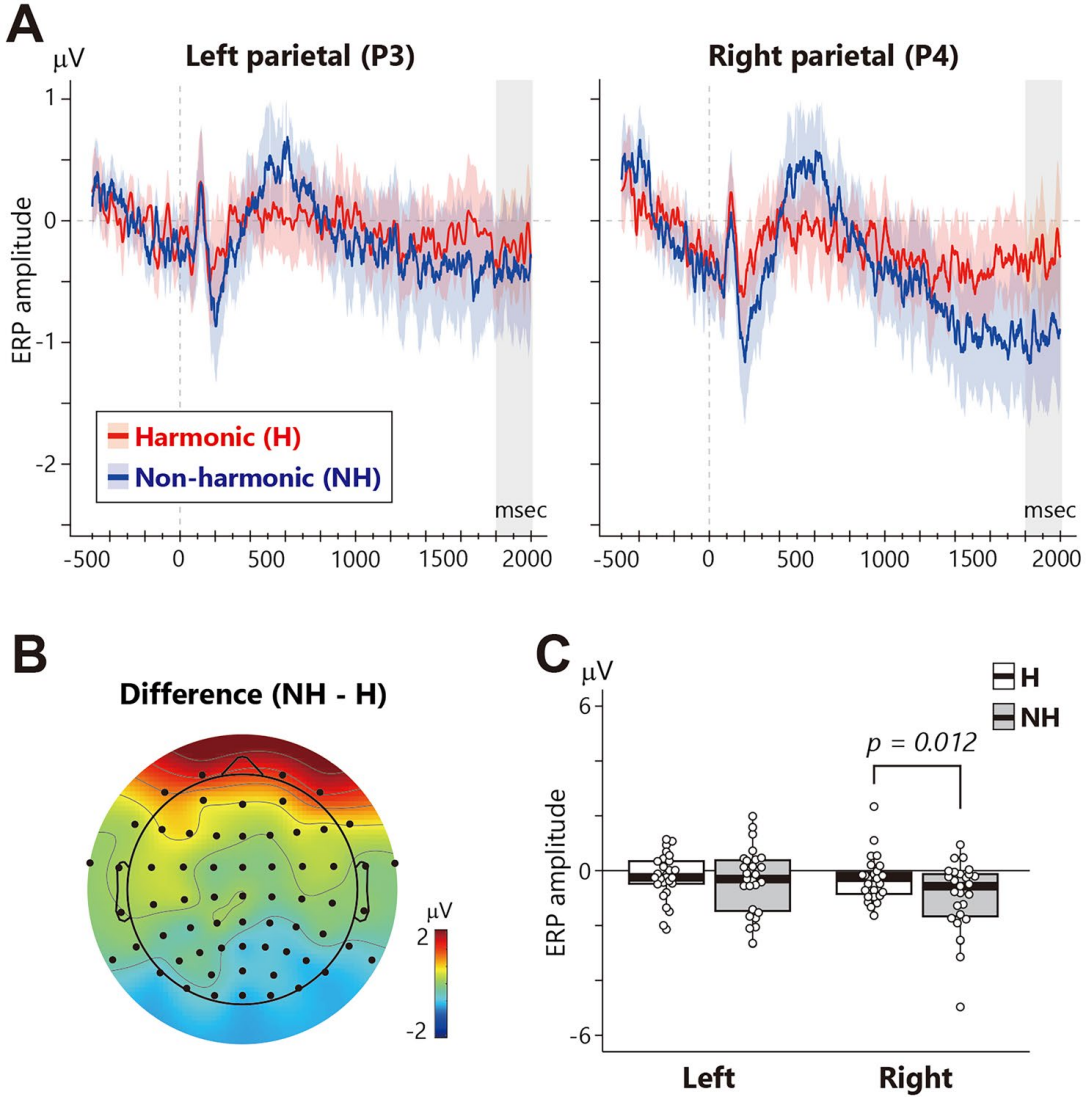
Waveform, topography, and mean amplitude of the SPN component. **(A)** SPN waveform for the third chord at P3 (left parietal) and P4 (right parietal) electrodes. The zero on the x-axis represents the onset of the third chord. Thin colored red and blue represent the regression line and 95% confidence interval, and the grey bar indicates the time range of SPN (1800–2000 ms). **(B)** Topographic map showing the difference between non-harmonic (NH) and harmonic (H) conditions in the time range of SPN. **(C)** Mean amplitude of SPN. The box-and-whisker plot displays the median, lower/higher quantile, and minimum/maximum of individual data (depicted as white circles).

Regarding the SPN, the right parietal electrode (P4) showed a late negative shift for both harmonic and non-harmonic conditions. In contrast, the left parietal electrode (P3) exhibited only a slight shift (Fig. 3A). The difference between non-harmonic and harmonic conditions on the topography map revealed a larger posterior negativity for non-harmonic condition (Fig. 3B). A two-way ANOVA of SPN amplitude, considering the factors of laterality (left and right) and harmony (harmonic and non-harmonic), showed a main effect of laterality (*F*_1, 26_ = 6.51, *p* = 0.017, partial *η*^2^ = 0.20) and a significant interaction (*F*_1, 26_ = 4.39, *p* = 0.046, partial *η*^2^ = 0.14). A simple effect analysis revealed that the right parietal electrode had a larger SPN for the non-harmonic condition than the harmonic condition (*F*_1, 26_ = 7.24, *p* = 0.012, partial *η*^2^ = 0.22; Fig. 3C). However, further analysis using Spearman’s rank correlation coefficient did not find a significant correlation between prediction certainty and SPN amplitude (left: *rho* = 0.18, *p* = 0.200; right: *rho* = 0.19, *p* = 0.178). These findings suggest that while the SPN in the right parietal area is sensitive to harmonic prediction, subjective certainty may not play a significant role in this response.

**Figure 4:**
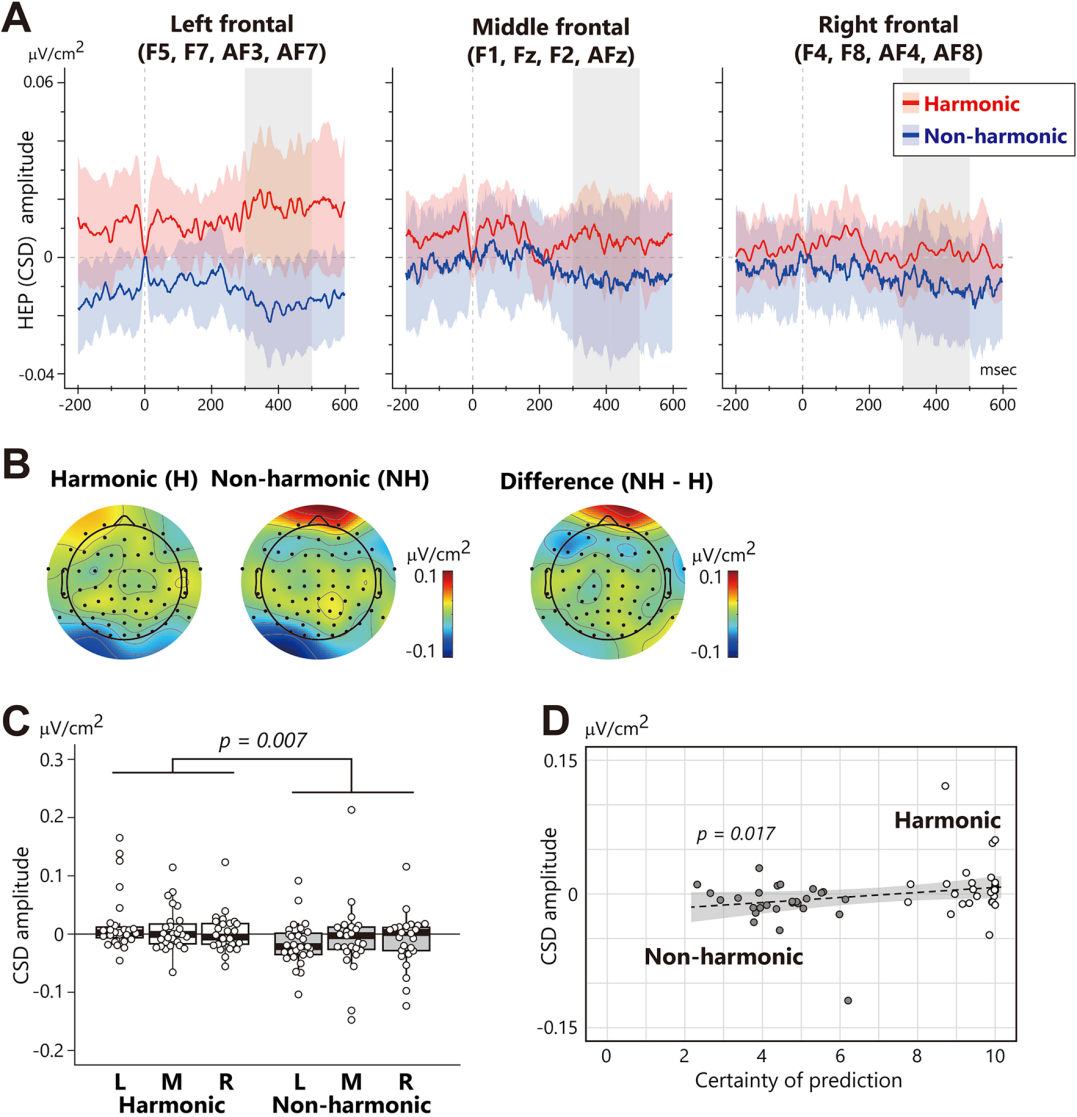
Waveform, topography, and mean amplitude of the HEP component. **(A)** CSD-transformed HEP waveforms at left frontal (F5, F7, AF3, and AF7), middle frontal (F1, Fz, F2, and AFz), and right frontal (F4, F8, AF4, and AF8) electrodes. The zero on the x-axis represents the onset of the R peak in ECG. Thin colored red and blue represent the regression line and 95% confidence interval, and the grey bar indicates the time range of HEP (300–500 ms). **(B)** Topographic map of the harmonic condition (H), non-harmonic condition (NH), and the difference between them (non-harmonic minus harmonic) in the time range of HEP. **(C)** Mean amplitude of CSD-transformed HEP. L, M, and R represent the left, middle, and right frontal regions, respectively. Each participant’s data is plotted as a white circle. **(D)** Scatter plot of the ratings of prediction certainty (x-axis) and the mean amplitude of CSD-transformed HEP for the third chord (y-axis). Each participant’s data is plotted as a grey (non-harmonic condition) and white (harmonic condition) circle. The dotted line and grey area represent the regression line and 95% confidence interval, respectively.

The HEP analysis displays the CSD-transformed HEP waveform in the frontal region and topographical map within the 300–500 ms time window after the R peaks in Fig. 4A,B. The difference between the CSD maps for harmonic and non-harmonic conditions showed a distinct effect in the frontal area (Fig. 4B right). A two-way ANOVA of the mean HEP amplitude revealed a main effect of harmony (*F*_1, 26_ = 8.74, *p* = 0.007, partial *η*^2^ = 0.25), indicating a larger HEP for the non-harmonic condition than the harmonic condition (Fig. 4C). To explore further the relationship between HEP and prediction certainty, Spearman’s rank correlation coefficient was calculated using the mean HEP amplitude across three regions and subjective ratings. This analysis found a significant positive correlation between HEP amplitude and prediction certainty (Fig. 4D: *rho* = 0.32, *p* = 0.017). These results suggest that the HEP amplitude becomes larger (more negative) in response to decreased subjective certainty in chord prediction.

As indicated by Fig. 4A, there appears to be a discrepancy in the baseline between conditions, which may contribute to the observed HEP difference. To address this, we calculated the difference between the mean HEP amplitude (from 300 to 500 ms) and the baseline activity (from − 200 to − 100 ms), and performed a post-hoc two-way ANOVA with harmony and ROIs as factors. Even after adjusting for the baseline difference, this analysis still revealed a significant main effect of harmony (*F*_1, 26_ = 5.27, *p* = 0.030, partial *η*^2^ = 0.17), affirming that the HEP difference between conditions remains significant.

In a post hoc analysis, we also calculated the Pearson correlation coefficient between the SPN amplitude at the P4 electrode and the mean HEP amplitude across three ROIs. This analysis did not produce significant results (*r* = 0.09, *p* = 0.511), indicating no interaction between SPN and HEP.

**Figure 5:**
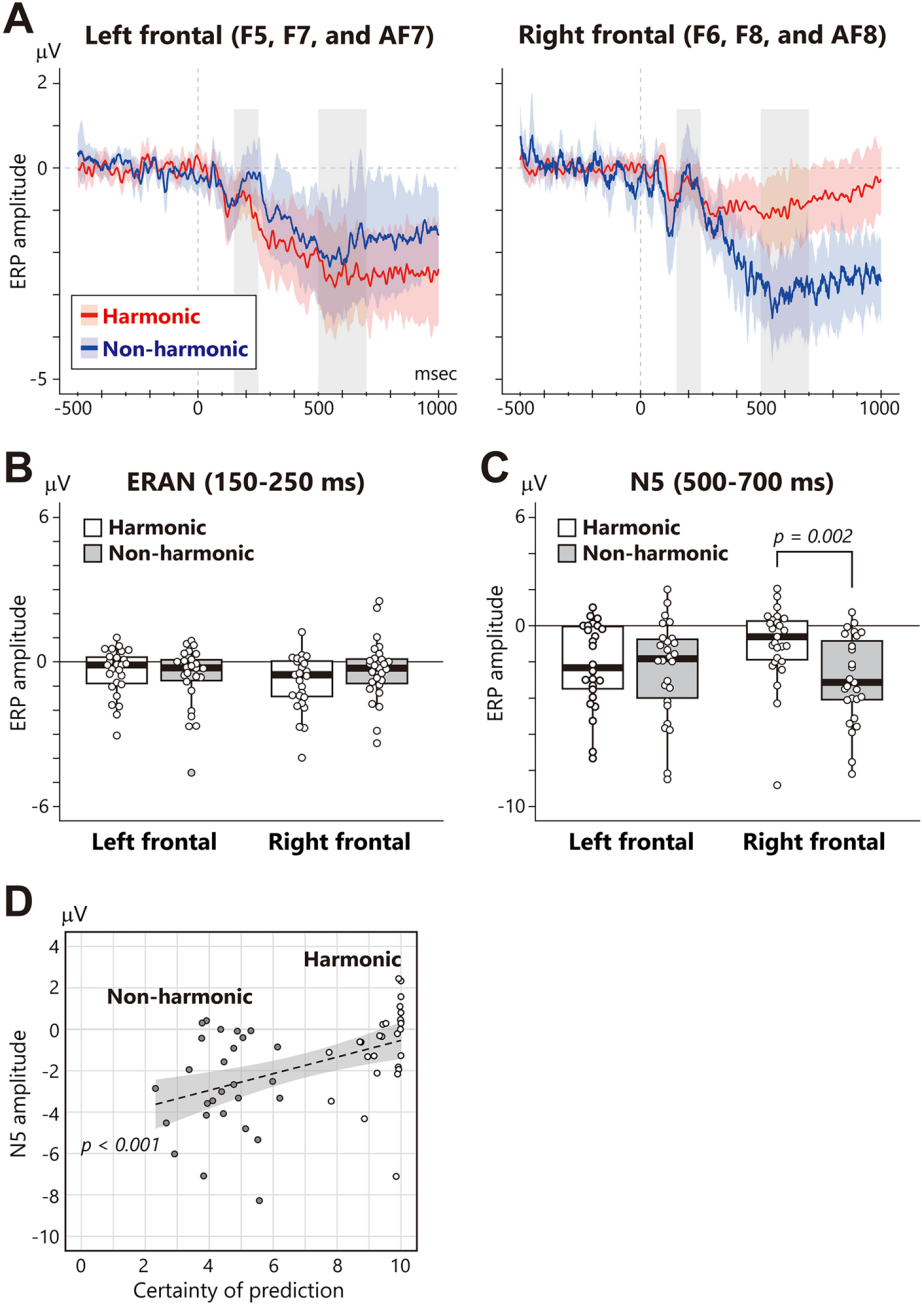
Waveform and mean amplitude of ERP components for the final chord. **(A)** ERP waveforms at right frontal (F6, F8, and AF8) electrodes. The zero on the x-axis represents the onset of the final chord. Thin colored red and blue represent the regression line and 95% confidence interval, and the grey bars indicate the time range of ERAN (150–250 ms) and N5 (500–700 ms). **(B)** Mean amplitude of ERAN. The box-and-whisker plot displays the median, lower/higher quantile, and minimum/maximum of individual data (depicted as white circles). **(C)** Mean amplitude of N5. The box-and-whisker plot displays the median, lower/higher quantile, and minimum/maximum of individual data (depicted as white circles). **(D)** Scatter plot of the ratings of prediction certainty (x-axis) and the mean amplitude of N5 (y-axis). Each participant’s data is plotted as a grey (non-harmonic condition) and white (harmonic condition) circle. The dotted line and grey area represent the regression line and 95% confidence interval, respectively.

To further examine the relationship between SPN/HEP and brain potentials for subsequent chords, ERAN and N5 for the final chord were analyzed. Figure 5A exhibits the mean waveform of the left and right frontal electrodes (left hemisphere: F5, F7, and AF7; right hemisphere: F6, F8, and AF8). The mean amplitude of ERAN and N5 were separately analyzed using two-way ANOVAs with the factors of harmony (harmonic and non-harmonic) and laterality (left hemisphere and right hemisphere). Although ERAN did not demonstrate a significant difference between the conditions (Fig. 5B), N5 exhibited a significant interaction (*F*_1, 26_ = 9.67, *p* = 0.005, partial *η*^2^ = 0.27). A simple effect analysis revealed that N5 in the non-harmonic condition was larger than that in the harmonic condition in the right frontal area (Fig. 5C: *F*_1, 26_ = 12.43, *p* = 0.002, partial *η*^2^ = 0.32). Additionally, a correlation analysis between N5 in the right frontal area and prediction certainty showed a significant correlation (Fig. 5D: *rho* = 0.51, *p* < 0.001), indicating that N5 was larger when the predictions were not accurate.

**Figure 6:**
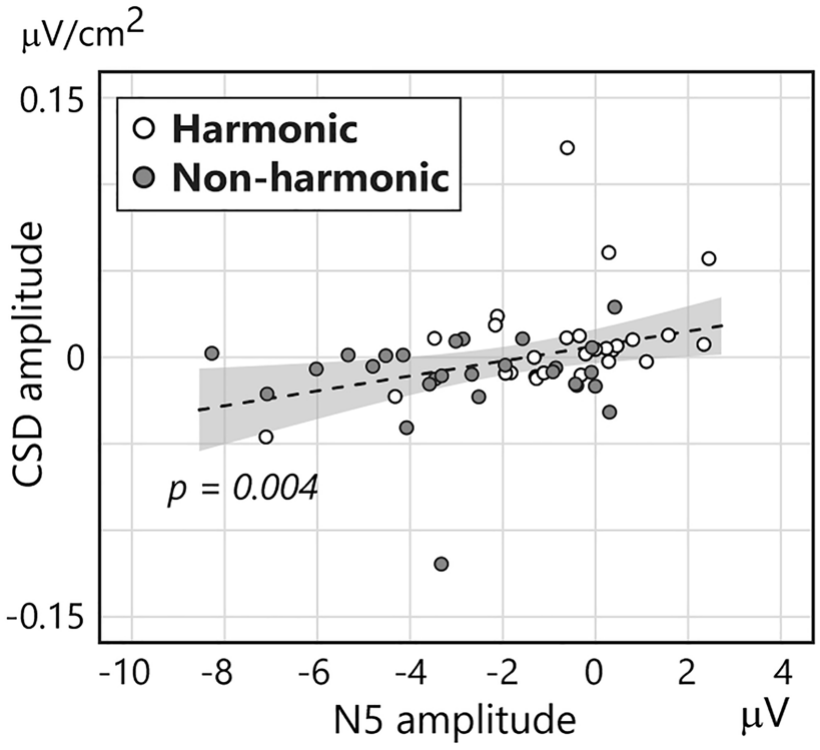
Scatter plot of the mean amplitude of N5 (x-axis) and CSD-transformed HEP (y-axis). Each participant’s data are plotted as a grey (non-harmonic condition) and white (harmonic condition) circle. The dotted line and grey area represent the regression line and 95% confidence interval, respectively.

The Pearson correlation coefficient was calculated in a post hoc analysis to explore the relationship between SPN/HEP and N5. This analysis showed a significant positive correlation between HEP for the third chord and N5 for the final chord (Fig. 6: *r* = 0.34, *p* = 0.011). However, there was no correlation between SPN at P4 and N5 (*r* = 0.10, *p* = 0.465). These results suggest that N5 is linked to HEP but not to SPN.

**Figure 7:**
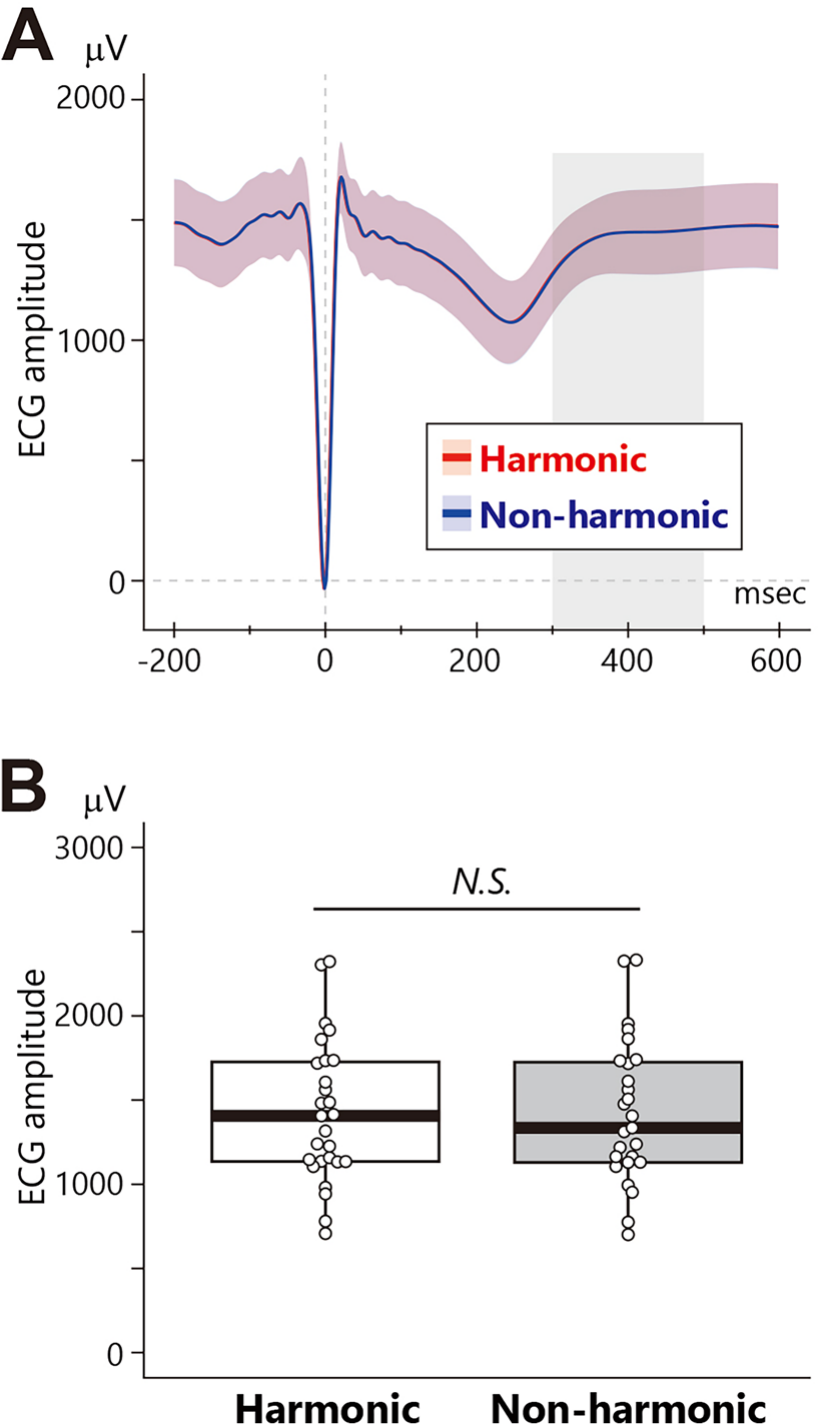
Waveform and mean amplitude of ECG for the third chord. **(A)** ECG waveforms. Thin red and blue represent the 95% confidence interval, and the grey bar indicates the HEP time range (300–500 ms). The zero on the x-axis represents the onset of the R peak. Note that the ECG waveform for the harmonic and non-harmonic conditions almost completely overlap. **(B)** The mean amplitude of ECG in the HEP time range (300–500 ms). The box-and-whisker plot displays the median, lower/higher quantile, and minimum/maximum of individual data (depicted as white circles).

Finally, to rule out the possibility that the observed HEP differences between conditions were influenced by artifactual effects in the ECG, we conducted similar preprocessing and analyses for ECG data as with HEP. The ECG waveform displayed a distinct QRS complex and T wave (Fig. 7A). A paired *t*-test using the mean amplitude of ECG within the 300–500 ms time window while participants listened to the third chord did not yield a significant difference (Fig. 7B: *t*_26_ = 0.17, *p* = 0.867, *r* = 0.03). This result suggests that there was no ECG difference that might contribute to the observed HEP difference between the conditions.

## Discussion

In this study, we investigated the relationship between the certainty of chord prediction and the neural correlates of prediction, specifically focusing on the SPN and HEP components. We found that 1) the SPN was larger for the non-harmonic condition compared to the harmonic condition, 2) the HEP was also larger for the non-harmonic condition compared to the harmonic condition and correlated with the ratings of harmonic certainty, 3) the N5 was larger for the non-harmonic condition and correlated with the ratings of harmonic certainty and HEP amplitude. These results suggest that the relationship between chord prediction certainty and neural responses was more pronounced for the HEP than the SPN.

Our observation of an increased SPN amplitude at the right parietal electrode for the non-harmonic condition aligns with previous studies that showed a larger SPN in unpredictable contexts^31,39,40^. SPN can be divided into an early phase with a flat waveform in amplitude around prefrontal and precentral areas and a late phase with a steep waveform in amplitude towards the target stimulus around the parietal area^34,66,86,87^. The early phase of SPN has been discussed in terms of attentional control of sensory processing, while the late phase has been discussed in terms of the emotionality of a target stimulus^34,88^. Since harmonically appropriate chord progressions elicit pleasantness and activate emotion-related brain structures like the amygdala^89–96^, we suggest that SPN results may reflect the difference in emotionality between chord sequences with and without harmonicity. However, as we did not evaluate the emotional aspect of chord prediction in this study, this speculation warrants further investigation in future studies.

While we discovered a larger SPN in unpredictable contexts, we did not find a correlation between SPN and prediction certainty. This result seems inconsistent with several previous SPN studies that demonstrated an association between the uncertainty of prediction and SPN amplitude^31,34,35,39–41^. This discrepancy may arise from differences in task design and analysis. Prior studies manipulated the probability of occurrence of a target stimulus (e.g., 0%, 50%, and 100%), and their analyses were grounded in these probability values. By contrast, our study did not control for chord occurrence probability, and our analysis was founded on the participants’ subjective ratings of prediction certainty. Given the randomness of stimulus presentation, individual difference could emerge regarding which chord succeeded another, potentially diluting the correlation due to biased presentation. To address this concern, we are currently designing the next phase of research to incorporate objective control of chord occurrence probability based on Western music theory.

In addition to SPN, our results revealed that the observed HEP differences between harmonic and non-harmonic conditions suggest that HEP may serve as a neural marker of harmonic prediction in music. This result is in line with previous studies that showed a significant relationship between pre-stimulus HEP and sensory processing^59,61–63^ and supports the hypothesis that interoceptive signals, as reflected by HEP, guide chord predictions. The significant correlation between HEP amplitude and participants’ subjective certainty in their chord predictions highlights the importance of considering individual differences in prediction-related neural responses. This finding emphasizes that HEP can be associated with chord (un) predictability and reflect the subjective evaluation of those predictions. Given the possibility that a musical context develops progressively throughout a chord sequence, it is conceivable that HEP amplitude might also increase gradually as this context becomes more firmly established. This is interesting idea and worth investigating in future studies.

Our hypothesis regarding the HEP is also supported by the significant relationship observed between HEP and N5. N5 is typically seen following ERAN, peaking around 500 ms after the onset of a chord^25,85,97^. While our study, along with some others, found a right hemisphere dominance for N5^27,85^, additional research has identified bilateral negativity in the frontal area^16,25,26^. The functional significance of this laterality difference remains to be fully understood, but N5 is generally interpreted as reflecting the integration of chords into a musical context. For instance, Koelsch and colleagues observed that in a harmonically appropriate chord progression, the N5 amplitude decreased as the musical context unfolded: the amplitude for the first chord was larger than that for subsequent chords. This pattern suggests that less harmonic integration is required as the progression continues. In the non-harmonic condition of our study, predicting the upcoming chord was challenging, as was integrating the presented chords into a specific musical context. Therefore, HEP and N5 might be correlated, reflecting the interplay between chord (un)predictability and the difficulty of integrating chords within a musical context.

A variety of factors could potentially influence the association between HEP and chord predictability, making it challenging to draw definitive conclusions from our results. Previous studies have shown that HEP is sensitive to psychological factors, such as motivation or arousal^98–100^. Predicting chords in the harmonic condition was substantially easier than in the non-harmonic condition, as evidenced by the ceiling effect observed in the subjective ratings shown in Fig. 1C. This could possibly decrease a participant’s motivation and arousal levels. While our study was not designed to evaluate these aspects, considering that arousal induction has been found to modulate HEP amplitude^99^, it would be worth examining the impact of these factors in future research.

Even though ERAN and N5 are often observed together in previous studies, we found an interesting functional distinction between them. Koelsch et al. (2000) interpreted ERAN and N5 as detecting the violation of a prediction based on a musical context and representing the integration process of chords within a musical context, respectively. This interpretation aligns well with our findings. In our experiment, we used a random sequence of chords as the non-harmonic condition, making it difficult to establish a robust musical context based on the first three chords. Larger N5 in the non-harmonic condition should reflect the stronger activation of the integration process to understand a chord sequence based on the implicit knowledge of harmony. Additionally, the participants could only rely on the third chord to predict the final chord in the non-harmonic condition. This would only create a weak musical context, which might lead to a weaker impact on the violation of prediction and a smaller ERAN. As a result, the integration process in the non-harmonic condition might have been activated and elicited N5, while the detection process of violation, reflected by ERAN, did not function as effectively. This idea could account for the observed functional differences between ERAN and N5 in our study. This explanation can be potentially confirmed in future studies that control chord predictability using statistical learning or other methods. This addition to our findings would help provide a more comprehensive understanding of the functional differences between ERAN and N5 in musical contexts.

There are some methodological limitations worth mentioning. First, since we did not assess the emotional aspects of subjective ratings, interpreting SPN and HEP in relation to emotion or arousal is speculative. Second, the similarity between the first and final chords, as well as the limited number of chord progressions in the harmonic condition, might have allowed participants to learn the progression within only a few repetitions. This learning effect potentially affected our results. Third, the non-harmonic condition was generated by randomly selecting chords, with no objective control for prediction certainty. Although correlation with subjective ratings were found, employing objective measures of prediction certainty, such as statistical learning or other methods, would provide a more robust evaluation of the effect. Fourth, pitch probability and tonal hierarchies are important parameters to develop a musical context, but our randomized presentation approach did not allow us for control over these factors. Exploring the impact of these parameters on harmonic prediction and associated ERPs would be valuable. These limitations should be considered when interpreting our findings and could be addressed in future studies.

In summary, our study investigated the relationship between SPN and HEP and the subjective certainty of chord prediction in harmonic and non-harmonic conditions. We found that HEP amplitude was significantly correlated with the subjective certainty of chord predictions and N5, suggesting its potential role as a neural marker of harmonic prediction. However, SPN did not significantly correlate with subjective certainty, possibly reflecting the role of emotional differences in the detection process rather than certainty levels.

Our results also revealed functional distinctions between ERAN and N5, which have often been observed together in previous studies. In our experiment, the integration process might have been activated, while the detection process did not function effectively. This is possibly due to the difficulty in generating a robust musical context based on the random sequence of chords. Although several factors, such as motivation or arousal, could potentially explain the relationship between HEP and chord predictability, our findings have some limitations. Future studies should address these limitations and further explore the underlying mechanisms of the observed relationship, as well as the potential interaction between interoceptive and exteroceptive processing in cognitive processes related to music perception and prediction.

## Data Availability

The raw data supporting the conclusions of this manuscript will be made available from the corresponding author on reasonable request.

## References

[1] Friston K, Kilner J, Harrison L (2006). A free energy principle for the brain. J. Physiol. Paris.

[2] Clark A (2013). Whatever next? Predictive brains, situated agents, and the future of cognitive science. Behav. Brain Sci..

[3] Koelsch S, Vuust P, Friston K (2019). Predictive processes and the peculiar case of music. Trends Cogn. Sci..

[4] Vuust P, Heggli OA, Friston KJ, Kringelbach ML (2022). Music in the brain. Nat. Rev. Neurosci..

[5] Pearce MT, Wiggins GA (2006). Expectation in melody: The influence of context and learning. Music Percept..

[6] Schmuckler, M. A. Expectation in music: Investigation of melodic. *Music Percept.* (2006).

[7] Thompson WF, Cuddy LL, Plaus C (1997). Expectancies generated by melodic intervals: evaluation of principles of melodic implication in a melody-completion task. Percept. Psychophys..

[8] Carlsen JC (1981). Some factors which influence melodic expectancy. Psychomusicol. J. Res. Music Cognit..

[9] Schmuckler MA (1989). Expectation in music: Investigation of melodic and harmonic processes. Music Percept..

[10] Steinbeis N, Koelsch S, Sloboda JA (2006). The role of harmonic expectancy violations in musical emotions: Evidence from subjective, physiological, and neural responses. J. Cogn. Neurosci..

[11] Cheung VKM (2019). Uncertainty and surprise jointly predict musical pleasure and amygdala, hippocampus, and auditory cortex activity. Curr. Biol..

[12] Patel AD (2010). Music, Language, and the Brain.

[13] Koelsch S (2001). Differentiating ERAN and MMN: An ERP study. Neuroreport.

[14] Koelsch S, Jentschke S, Sammler D, Mietchen D (2007). Untangling syntactic and sensory processing: An ERP study of music perception. Psychophysiology.

[15] Koelsch S (2009). Music-syntactic processing and auditory memory: Similarities and differences between ERAN and MMN. Psychophysiology.

[16] Koelsch S, Schmidt B-H, Kansok J (2002). Effects of musical expertise on the early right anterior negativity: An event-related brain potential study. Psychophysiology.

[17] Koelsch S, Kilches S, Steinbeis N, Schelinski S (2008). Effects of unexpected chords and of performer’s expression on brain responses and electrodermal activity. PLoS ONE.

[18] Janata P (1995). ERP measures assay the degree of expectancy violation of harmonic contexts in music. J. Cogn. Neurosci..

[19] Jentschke S, Koelsch S, Sallat S, Friederici AD (2008). Children with specific language impairment also show impairment of music-syntactic processing. J. Cogn. Neurosci..

[20] Brattico E, Jacobsen T, De Baene W, Glerean E, Tervaniemi M (2010). Cognitive versus affective listening modes and judgments of music—An ERP study. Biol. Psychol..

[21] Müller M, Höfel L, Brattico E, Jacobsen T (2010). Aesthetic judgments of music in experts and laypersons—An ERP study. Int. J. Psychophysiol..

[22] Kalda T, Minati L (2012). Detecting scale violations in absence of mismatch requires music-syntactic analysis: A further look at the early right anterior negativity (ERAN). Brain Topogr..

[23] Tervaniemi M, Tupala T, Brattico E (2012). Expertise in folk music alters the brain processing of Western harmony. Ann. N. Y. Acad. Sci..

[24] Bianco R (2016). Neural networks for harmonic structure in music perception and action. Neuroimage.

[25] Koelsch S, Gunter T, Friederici AD, Schröger E (2000). Brain indices of music processing: “Nonmusicians” are musical. J. Cogn. Neurosci..

[26] Koelsch S, Rohrmeier M, Torrecuso R, Jentschke S (2013). Processing of hierarchical syntactic structure in music. Proc. Natl. Acad. Sci. U.S.A..

[27] Koelsch S, Schroger E, Gunter TC (2002). Music matters: Preattentive musicality of the human brain. Psychophysiology.

[28] Koelsch S (2011). Towards a neural basis of processing musical semantics. Phys. Life Rev..

[29] Brunia CHM, Hackley SA, van Boxtel GJM, Kotani Y, Ohgami Y (2011). Waiting to perceive: Reward or punishment?. Clin. Neurophysiol..

[30] Böcker KB, Brunia CH, van den Berg-Lenssen MM (1994). A spatiotemporal dipole model of the stimulus preceding negativity (SPN) prior to feedback stimuli. Brain Topogr..

[31] Catena A (2012). The brain network of expectancy and uncertainty processing. PLoS ONE.

[32] Kotani Y (2015). Source analysis of stimulus-preceding negativity constrained by functional magnetic resonance imaging. Biol. Psychol..

[33] Ohgami Y, Kotani Y, Arai J-I, Kiryu S, Inoue Y (2014). Facial, verbal, and symbolic stimuli differently affect the right hemisphere preponderance of stimulus-preceding negativity. Psychophysiology.

[34] Ohgami Y (2021). Voice, rhythm, and beep stimuli differently affect the right hemisphere preponderance and components of stimulus-preceding negativity. Biol. Psychol..

[35] Brunia CHM, van Boxtel GJM (2004). Anticipatory attention to verbal and non-verbal stimuli is reflected in a modality-specific SPN. Exp. Brain Res..

[36] Ono K, Hashimoto J, Hiramoto R, Sasaoka T, Yamawaki S (2021). Modulatory effects of prediction accuracy on electroencephalographic brain activity during prediction. Front. Hum. Neurosci..

[37] Brown CA, Seymour B, Boyle Y, El-Deredy W, Jones AKP (2008). Modulation of pain ratings by expectation and uncertainty: Behavioral characteristics and anticipatory neural correlates. Pain.

[38] Brunia CH (1988). Movement and stimulus preceding negativity. Biol. Psychol..

[39] Fuentemilla L (2013). Electrophysiological correlates of anticipating improbable but desired events. Neuroimage.

[40] Morís J, Luque D, Rodríguez-Fornells A (2013). Learning-induced modulations of the stimulus-preceding negativity. Psychophysiology.

[41] Ohgami Y (2006). Effects of monetary reward and punishment on stimulus-preceding negativity. Psychophysiology.

[42] León-Cabrera P, Rodríguez-Fornells A, Morís J (2017). Electrophysiological correlates of semantic anticipation during speech comprehension. Neuropsychologia.

[43] León-Cabrera P, Flores A, Rodríguez-Fornells A, Morís J (2019). Ahead of time: Early sentence slow cortical modulations associated to semantic prediction. Neuroimage.

[44] Fiveash A, Thompson WF, Badcock NA, McArthur G (2018). Syntactic processing in music and language: Effects of interrupting auditory streams with alternating timbres. Int. J. Psychophysiol..

[45] Koelsch S (2004). Music, language and meaning: Brain signatures of semantic processing. Nat. Neurosci..

[46] Sammler D (2013). Co-localizing linguistic and musical syntax with intracranial EEG. Neuroimage.

[47] Strigo IA, Craig ADB (2016). Interoception homeostatic emotions and sympathovagal balance. Philos. Trans. R. Soc. Lond. B Biol. Sci..

[48] Craig AD (2002). How do you feel? Interoception: The sense of the physiological condition of the body. Nat. Rev. Neurosci..

[49] Barrett LF, Simmons WK (2015). Interoceptive predictions in the brain. Nat. Rev. Neurosci..

[50] Seth AK, Friston KJ (2016). Active interoceptive inference and the emotional brain. Philos. Trans. R. Soc. Lond. B Biol. Sci..

[51] Marshall AC, Gentsch A, Schütz-Bosbach S (2018). The Interaction between Interoceptive and Action States within a Framework of Predictive Coding. Front. Psychol..

[52] Ambrosini E, Finotti G, Azevedo RT, Tsakiris M, Ferri F (2019). Seeing myself through my heart: Cortical processing of a single heartbeat speeds up self-face recognition. Biol. Psychol..

[53] Galvez-Pol A, McConnell R, Kilner JM (2020). Active sampling in visual search is coupled to the cardiac cycle. Cognition.

[54] Salomon R (2016). The insula mediates access to awareness of visual stimuli presented synchronously to the heartbeat. J. Neurosci..

[55] Canales-Johnson A (2015). Auditory feedback differentially modulates behavioral and neural markers of objective and subjective performance when tapping to your heartbeat. Cereb. Cortex.

[56] Motyka P (2019). Interactions between cardiac activity and conscious somatosensory perception. Psychophysiology.

[57] Walker BB, Sandman CA (1982). Visual evoked potentials change as heart rate and carotid pressure change. Psychophysiology.

[58] van Elk M, Lenggenhager B, Heydrich L, Blanke O (2014). Suppression of the auditory N1-component for heartbeat-related sounds reflects interoceptive predictive coding. Biol. Psychol..

[59] Al E (2020). Heart-brain interactions shape somatosensory perception and evoked potentials. Proc. Natl. Acad. Sci. U.S.A..

[60] Coll M-P, Hobson H, Bird G, Murphy J (2021). Systematic review and meta-analysis of the relationship between the heartbeat-evoked potential and interoception. Neurosci. Biobehav. Rev..

[61] Al E, Iliopoulos F, Nikulin VV, Villringer A (2021). Heartbeat and somatosensory perception. Neuroimage.

[62] Marshall AC, Gentsch A, Schütz-Bosbach S (2020). Interoceptive cardiac expectations to emotional stimuli predict visual perception. Emotion.

[63] Park H-D, Correia S, Ducorps A, Tallon-Baudry C (2014). Spontaneous fluctuations in neural responses to heartbeats predict visual detection. Nat. Neurosci..

[64] Okubo M, Suzuki H, Nicholls MER (2014). A Japanese version of the FLANDERS handedness questionnaire. Shinrigaku Kenkyu.

[65] Delorme A, Makeig S (2004). EEGLAB: An open source toolbox for analysis of single-trial EEG dynamics including independent component analysis. J. Neurosci. Methods.

[66] Brunia CH, Damen EJ (1988). Distribution of slow brain potentials related to motor preparation and stimulus anticipation in a time estimation task. Electroencephalogr. Clin. Neurophysiol..

[67] Winkler I (2014). Robust artifactual independent component classification for BCI practitioners. J. Neural Eng..

[68] Winkler I, Haufe S, Tangermann M (2011). Automatic classification of artifactual ICA-components for artifact removal in EEG signals. Behav. Brain Funct..

[69] Park H-D, Blanke O (2019). Heartbeat-evoked cortical responses: Underlying mechanisms, functional roles, and methodological considerations. Neuroimage.

[70] Lopez-Calderon J, Luck SJ (2014). ERPLAB: An open-source toolbox for the analysis of event-related potentials. Front. Hum. Neurosci..

[71] Kayser J, Tenke CE (2015). Issues and considerations for using the scalp surface Laplacian in EEG/ERP research: A tutorial review. Int. J. Psychophysiol..

[72] Kamarajan C, Pandey AK, Chorlian DB, Porjesz B (2015). The use of current source density as electrophysiological correlates in neuropsychiatric disorders: A review of human studies. Int. J. Psychophysiol..

[73] Pollatos O, Herbert BM, Mai S, Kammer T (2016). Changes in interoceptive processes following brain stimulation. Philos. Trans. R. Soc. Lond. B Biol. Sci..

[74] Park H-D (2016). Transient modulations of neural responses to heartbeats covary with bodily self-consciousness. J. Neurosci..

[75] Gentsch A, Sel A, Marshall AC, Schütz-Bosbach S (2019). Affective interoceptive inference: Evidence from heart-beat evoked brain potentials. Hum. Brain Mapp..

[76] Fukushima H, Terasawa Y, Umeda S (2011). Association between interoception and empathy: Evidence from heartbeat-evoked brain potential. Int. J. Psychophysiol..

[77] Montoya P, Schandry R, Müller A (1993). Heartbeat evoked potentials (HEP): Topography and influence of cardiac awareness and focus of attention. Electroencephalogr. Clin. Neurophysiol..

[78] Pollatos O, Schandry R (2004). Accuracy of heartbeat perception is reflected in the amplitude of the heartbeat-evoked brain potential: Heartbeat-evoked potential and heartbeat perception. Psychophysiology.

[79] Schandry R, Montoya P (1996). Event-related brain potentials and the processing of cardiac activity. Biol. Psychol..

[80] Pollatos O, Kirsch W, Schandry R (2005). Brain structures involved in interoceptive awareness and cardioafferent signal processing: A dipole source localization study. Hum. Brain Mapp..

[81] Dirlich G, Vogl L, Plaschke M, Strian F (1997). Cardiac field effects on the EEG. Electroencephalogr. Clin. Neurophysiol..

[82] Damen EJ, Brunia CH (1994). Is a stimulus conveying task-relevant information a sufficient condition to elicit a stimulus-preceding negativity?. Psychophysiology.

[83] Sammler D, Koelsch S, Friederici AD (2011). Are left fronto-temporal brain areas a prerequisite for normal music-syntactic processing?. Cortex.

[84] Garza Villarreal EA, Brattico E, Leino S, Ostergaard L, Vuust P (2011). Distinct neural responses to chord violations: A multiple source analysis study. Brain Res..

[85] Miranda RA, Ullman MT (2007). Double dissociation between rules and memory in music: An event-related potential study. Neuroimage.

[86] Hellwig S (2008). Slow cortical potentials in human aversive trace conditioning. Int. J. Psychophysiol..

[87] Seidel E-M (2015). Uncertainty during pain anticipation: The adaptive value of preparatory processes. Hum. Brain Mapp..

[88] Hopf JM, Mangun GR (2000). Shifting visual attention in space: An electrophysiological analysis using high spatial resolution mapping. Clin. Neurophysiol..

[89] Blood AJ, Zatorre RJ (2001). Intensely pleasurable responses to music correlate with activity in brain regions implicated in reward and emotion. Proc. Natl. Acad. Sci. U.S.A..

[90] Koelsch S, Fritz T, Schulze K, Alsop D, Schlaug G (2005). Adults and children processing music: An fMRI study. Neuroimage.

[91] Koelsch S, Fritz T, Cramon DYV, Müller K, Friederici AD (2006). Investigating emotion with music: an fMRI study. Hum. Brain Mapp..

[92] Koelsch S, Fritz T, Schlaug G (2008). Amygdala activity can be modulated by unexpected chord functions during music listening. Neuroreport.

[93] Salimpoor VN, Benovoy M, Larcher K, Dagher A, Zatorre RJ (2011). Anatomically distinct dopamine release during anticipation and experience of peak emotion to music. Nat. Neurosci..

[94] Salimpoor VN (2013). Interactions between the nucleus accumbens and auditory cortices predict music reward value. Science.

[95] Lehne M, Rohrmeier M, Koelsch S (2014). Tension-related activity in the orbitofrontal cortex and amygdala: An fMRI study with music. Soc. Cogn. Affect. Neurosci..

[96] Trost W (2014). Getting the beat: entrainment of brain activity by musical rhythm and pleasantness. Neuroimage.

[97] Koelsch S (2005). Neural substrates of processing syntax and semantics in music. Curr. Opin. Neurobiol..

[98] Weitkunat R, Schandry R (1990). Motivation and heartbeat evoked potentials. J. Psychophysiol..

[99] Luft CDB, Bhattacharya J (2015). Aroused with heart: Modulation of heartbeat evoked potential by arousal induction and its oscillatory correlates. Sci. Rep..

[100] Shao S, Shen K, Wilder-Smith EPV, Li X (2011). Effect of pain perception on the heartbeat evoked potential. Clin. Neurophysiol..

